# A Meta-Review of Spatial Transcriptomics Analysis Software

**DOI:** 10.3390/cells14141060

**Published:** 2025-07-10

**Authors:** Jessica Gillespie, Maciej Pietrzak, Min-Ae Song, Dongjun Chung

**Affiliations:** 1 Department of Biomedical Informatics, The Ohio State University, Columbus, OH 43210, USA; jessica.gillespie@osumc.edu (J.G.); maciej.pietrzak@osumc.edu (M.P.); 2 Comprehensive Cancer Center, The Ohio State University, Columbus, OH 43210, USA; 3 Division of Environmental Health Science, College of Public Health, The Ohio State University, Columbus, OH 43210, USA; song.991@osu.edu

**Keywords:** spatial transcriptomics, benchmarking, tissue architecture identification, spatially variable gene, cell–cell communication, deconvolution

## Abstract

Spatial transcriptomics combines gene expression data with spatial coordinates to allow for the discovery of detailed RNA localization, study development, investigating the tumor microenvironment, and creating a tissue atlas. A large range of spatial transcriptomics software is available, with little information on which may be better suited for particular datasets or computing environments. A review was conducted to detail the useful metrics when choosing appropriate software for spatial transcriptomics analysis. Specifically, the results from benchmarking studies that compared software across four key areas of spatial transcriptomics analysis (tissue architecture identification, spatially variable gene discovery, cell–cell communication analysis, and deconvolution) were assimilated into a single review that can serve as guidance when choosing potential spatial transcriptomics analysis software.

## 1. Introduction

From the initial decoding of the human genome, the fields of next-generation sequencing (NGS) and bioinformatics continue to rapidly expand in terms of both the available technologies and methods of analysis. While the ability to read DNA and RNA emerged early, advancements in the depth and breadth are still being made. Bulk RNA-seq provides a measure of gene expression in a sample, revealing valuable information about cellular activities [1,2]. This expression, however, is merely an average gene expression, as bulk RNA-seq produces only sample-level information. The next iteration of sequencing, single-cell RNA-seq (scRNA-seq), provides gene expression at the individual cell level but obscures the location of cells in a sample [3,4]. Gene expression in relation to tissue structure has become an increasingly important factor in the study of many diseases and conditions, and so the field of spatial transcriptomics (ST) emerged [5].

ST employs various methods of cellular dissection to capture and create a two-dimensional expression map of a sample [6]. This map can detail the gene expressions limited to a specific region of cells in the sample, can place into context the function of a cell in relation to the location, and can illuminate active cell communication happening across a region [7]. Because ST conserves the physical location of the RNA expression, the field has unique clinical applications, such as precisely mapping tumor heterogeneity and immune infiltration to better inform treatment and track progression. Embryonic development can be understood at a much deeper level than with bulk RNA or scRNA-seq. This information is gathered at a resolution ranging from 10s of cells down to the sub-cellular level [7,8].

ST analysis consists of many steps, but there are four that are frequently performed: tissue architecture identification, spatially variable gene (SVG) detection, cell–cell communication (CCC) analysis, and deconvolution. Tissue architecture identification combines gene expression profiles with spatial coordinates to group individual cells or cell spots, enabling the assignment of cell-type labels and other group analyses. SVG detection shows which genes’ expressions vary across a tissue sample in a statistically significant way. In cell–cell communication analysis, gene expression is compared with a ligand–receptor database while considering the spatial location of cells expressing either component to calculate the probability that two cells are interacting with one another. Finally, as some spatial sequencing methods are not at the single-cell resolution, deconvolution is needed to predict the proportion of cell types present in each spatial spot assayed.

Recently, a large range of software has been developed for the analysis of one or all of the mentioned steps. While individual papers exist for each software package, demonstrating their abilities, the datasets analyzed, hardware utilized, and quality metrics vary widely across publications. Comparing software head-to-head under controlled conditions provides a more accurate assessment, which is achieved in benchmarking studies and the basis for the systematic review given here. Specifically, we considered various factors for recommendations, although not with equal priority: accuracy; runtime; system requirements; programming language; and compatibility with the 10x Visium platform, a popular choice for ST sequencing.

## 2. Tissue Architecture Identification

Established gene expression profiles and the unique advantage of the physical location of cells of ST allow one to algorithmically “reassemble” tissue samples. Benchmarking analyses compared each software against real and simulated data with the established ground truth and ranked them by accuracy while also noting consistent performance between the datasets, runtimes, and resource requirements (Table 1).

Cheng et al. [35] explored 15 different software packages using seven real ST datasets accompanied by simulated gene expression and Hematoxylin and Eosin (H&E) data. Of note, they found options that did not use spatial coordinates or histological data (i.e., Seurat) were not necessarily disadvantaged without those data and better histology did not correlate with more accurate clustering. They also found little difference in the performance when the software had different algorithmic choices (i.e., Seurat-LV, SLM, LVM, Giotto-H, HM, LD). Another benchmark analysis assessed 16 software packages using 10 real ST datasets based on accuracy and how they handled spatial continuity (along with other analysis software not discussed here [36]). They tested the effect of random seeds where applicable and found the performance was associated with the provided random seed value. A final study benchmarked 13 packages across seven datasets, which notably compared the accuracy in-depth across not only data but also sample origin and spatial technology [37]. While some software performed well across all of the metrics, it was found that both the sample origin (tissue and patient) and spatial technology used to image the sample played large roles in the accuracy of the results.

Consolidating the accuracy results from the three benchmarks while also considering the runtime, necessary computer resources, and compatibility with 10x Visium data, the following software is suggested. BASS [11] and BayesSpace [12] were consistently in the top five in accuracy in all three benchmarks, although both scaled poorly with increased dataset size and BayesSpace did not perform well when given imaging data (as opposed to sequencing data). SpaGCN [38] was weak to a lack of spatial patterns but consistently performed quickly and well. Seurat [39] also showed high accuracy and good runtime and the advantage of not requiring histology data. A final option, STAGATE [28], was less accurate than those previously mentioned but still a solid choice if none of the others fit the desired workflow.

Some universally applicable observations were made by one or more of the benchmarking groups. All noted to some degree that the pre-processing employed by the software had a major effect on the accuracy while post-processing usually universally improved accuracy. For any methods that require a specific cluster number input by the user, the provided number could heavily influence the accuracy if it did not match the ground truth number of cell types present in the sample. Each benchmark also noted that the analysis performance was highly dataset dependent, meaning one software package could perform very well with brain tissue but struggle with spinal tissue with no general pattern to predict which software was the best choice for a given sample. Several authors postulated this fluctuation in performance could be a result of overfitting on the relatively small ST dataset collection currently available.

Another general observation was that no one particular algorithm type outperformed the others in clustering. While BASS and BayesSpace are both Bayesian-based (statistical), SpaGCN and Seurat are graph-based and performed nearly as well. The next best options STAGATE and CCST [13] (not suggested here for 10x Visium data) were deep learning-based, signifying the top recommendations were an equal mixture of available method types. This will not always hold true in other areas of ST analysis.

## 3. Spatially Variable Gene Discovery

Once counts are normalized in a sample, a popular next step is to find genes whose expression differs across physical space. Spatially variable genes (SVGs) can illuminate “regions” of cell activity that are not evident through histology or sequencing alone. While there are numerous options for SVG analysis, not many benchmarks have been performed yet (Table 2). Still, there are some helpful notes and patterns that can be gleaned from the available literature.

Li et al. [49] used simulated ground truth data with a variety of spatial patterns to mimic real-world data scenarios. They found noise played a large part in accurately determining the SVGs, as the performance of all software packages decreased with increased noise in the data. Highly variable gene (HVG) information is sometimes used as a feature for tissue architecture identification but can become much more powerful when combined with SVGs. A second benchmark by Chen et al. [50] evaluated SVG software by exploring the correlation between them and found that although the statistics were similar, the returned lists of SVGs had very little overlap. They also found that SVG ranking correlated positively with gene expression and that most packages had a high false discovery rate (FDR) when working with simulated, ground truth data.

Of the software benchmarked, SpatialDE2 [42] often topped the results in regard to accuracy, although it struggled with downsampling or high numbers of spatial locations and was slow and resource-heavy. SPARK-X [48], on the other hand, was consistently in the top three for accuracy and significantly faster and lighter on RAM than SpatialDE2. Likewise, SOMDE [47] was fast and light with a good FDR but suffered from low sensitivity. Finally, Moran’s I [51] was often second in rankings and had a unique approach due to its permutation-based algorithm. It is resource-light and performs well with sparse spatial locations; however, it suffers from a high FDR.

The SVG results varied as, once again, the available software seemed to be built for a specific sample type due to this being a new area of research and having relatively few datasets to test on. Across both benchmarks and all analysis methods, the SVG rankings correlated with gene expression, which created a possible source of universal bias. Overall, the available software had either a high sensitivity or high specificity but not both. Finally, the two major method types, graph-based and kernel-based, performed equally in all assessments.

## 4. Cell–Cell Communication Analysis

Cells “talk” to one another by sending chemical ligands that bind to receptors on other cells, setting in motion various biological pathways [52]. Some of these signals happen between adjacent (touching) cells, while others are sent to cells further away [53]. After the spatial location of cells is determined and their gene expression evaluated, a further step can be to determine whether cells are communicating with one another. Typically, this analysis is accomplished by finding ligand expression in a group of cells and the corresponding receptor in another [54,55,56]. Communication is assumed if matching ligand–receptor pairs are expressed from cells in the expected proximity befitting of the communication pair. With ST data, the proximity can be precisely calculated in a way that bulk or scRNA-seq cannot provide. Unfortunately, at the current time, there is no way to validate that the gene expression of ligand–receptor pairs guarantees communication and is a caveat of this analysis. However, CCC could be integrated with one of several methods to measure interaction by inducing fluorescence resonance energy transfers [57], which uses tagged donor/receiver molecules to explore interactions or support planar lipid bilayers [58], which allows for the imaging of protein interaction and organization.

Liu et al. [59] compared the concordance of ligand–receptor pairs returned by 16 CCC software packages with a simulated dataset and between the packages themselves. They created a metric, the distance enrichment score (DES), that uses the difference between the expected (from simulated datasets) and observed (from real datasets) spatial distance tendencies to determine the accuracy of the CCC analysis software (Table 3). CellChat [60], ICELLNET [61], and CellPhoneDB [62] all performed very well with most datasets. They were all fast and light on resources and had good consistency when given spatial data. CellChat also integrated regulatory information, which boosted its ability to identify ligand–receptor pairs. SingleCellSignalR [63] also performed well but was resource-heavy and had low precision. NicheNet [64] was a consistent second place with many datasets and uses a unique network-based method, while the other methods mentioned are statistics-based. NicheNet was very resource-light and handled sparse spatial data well but suffered from a high FDR.

In keeping with the theme from previous sections, CCC software accuracy was heavily dependent on the dataset analyzed. This was compounded in CCC by the fact many packages use completely different ligand–receptor databases from which to pull interactions. Two options may therefore return differing results merely due to the database packaged with them. Finally, it was noted that software that predicted more interactions had a higher recall (sensitivity), while those that returned fewer interactions had a higher precision (positive predictive value).

## 5. Deconvolution

As some ST technologies are not at the single-cell resolution, sequencing happens in a small section of the tissue called “spots”. Deconvolution predicts the proportion of cell types in the population of each sequencing spot. A number of software packages employing a variety of algorithms exist (Table 4) and were tested focusing on accuracy against real and simulated datasets while noting the runtime and resources required.

A first benchmark from Li et al. [86] showed that library preparation played a key role in the analysis results, noting the choice of RNA library preparation and sequencing platforms could affect the deconvolution due to differences in the gene expression profiles. Also, variation in the datasets between scRNA-seq and ST data for the same sample presented a problem, as the prior for this analysis assumed the cell populations were identical in both. Yan and Sun [87] explored sequencing depth in more detail than others and found most software held up well at various sequencing depths but suffered when the spot size became smaller. Data sparsity was found to greatly affect the performance of integration methods that predicted the spatial distribution of RNA transcripts in a final benchmark [88].

Cell2location [74] offered the highest accuracy of all the options but at the cost of heavy resource requirements and long runtimes. Tangram [81] was nearly as accurate, with a much lighter resource load, shorter runtime, and high proficiency when predicting the spatial distribution of transcripts. RCTD [77] steadily performed in the “top 5” ranking across all benchmarks and is offered as a solid alternative to the previously mentioned. A final method from Berglund et al. was noted in a benchmark as a deconvolution option that does not require scRNA-seq data. The results from this method are not favorable compared with others and the authors recommended to always use scRNA-seq data for deconvolution.

Unlike tissue architecture identification, deconvolution’s best performers were heavily biased towards probabilistic and deep learning-based methods (NMF-based, graph-based, and optimal transport-based did not do well comparatively). All benchmarks noted that normalization greatly affected the performance. Raw data counts were generally preferable to lognorm and scatternorm methods where available [86], normalization could vary in performance across different sample types [87], and raw spatial data worked best with either raw or normalized scRNA-seq data [88]. One benchmark mentioned EnDecon [89], a software package that integrates multiple deconvolution results to improve accuracy, and concluded that the increase in accuracy was marginal compared with well-tuned solitary options. Once again, there was wide variation in the software accuracy depending on the dataset, suggesting the overfitting of algorithms to specialized cases.

## 6. Computing Resource Requirements and Accuracy Metrics

Determining the best route for ST analysis requires considering several factors. First, fundamental concepts, such as the analysis accuracy or potential throughput capacity, must be considered across all software to determine an analysis timeframe and computer requirements. However, compatibility with the tissue sample under study and the choice of whether to normalize the data before running the analysis appear to be equally important factors in determining high-quality data.

**Figure 1:**
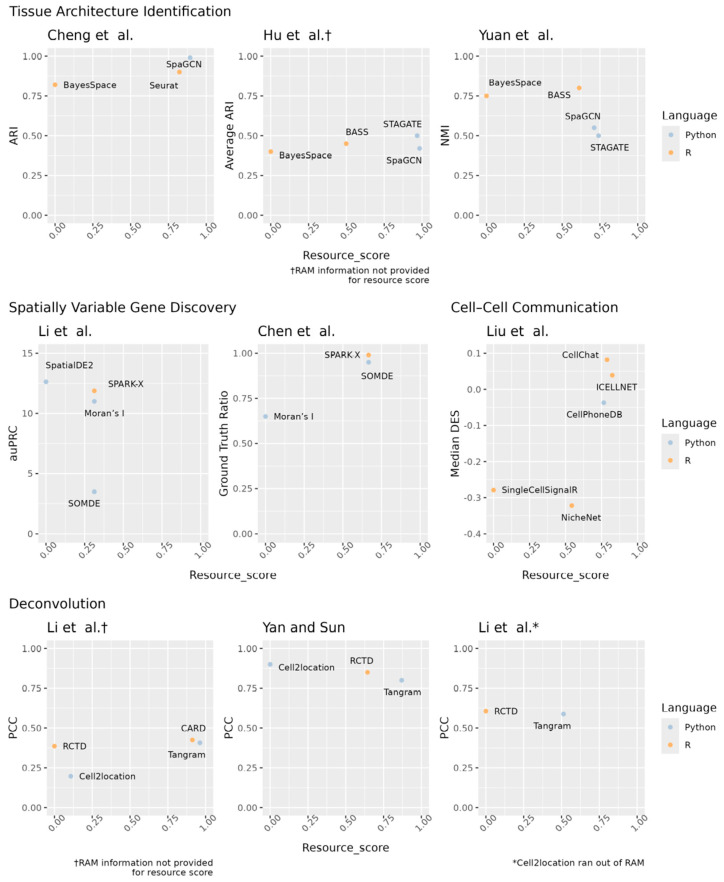
Visual comparison of software by benchmarking study using the accuracy (y-axis) and resource score (x-axis). The accuracy metrics were provided by each paper. The resource score is the average normalized time and RAM requirements subtracted from one. Higher values are better in all cases. Cheng et al. [35], Hu et al. [36], Yuan et al. [37], Li et al. [49], Chen et al. [50], Liu et al. [59], Li et al. [86], Yan and Sun [87], Li et al. [88]. † RAM information not provided for resource score. * Cell2location ran out of RAM.

**Figure 2:**
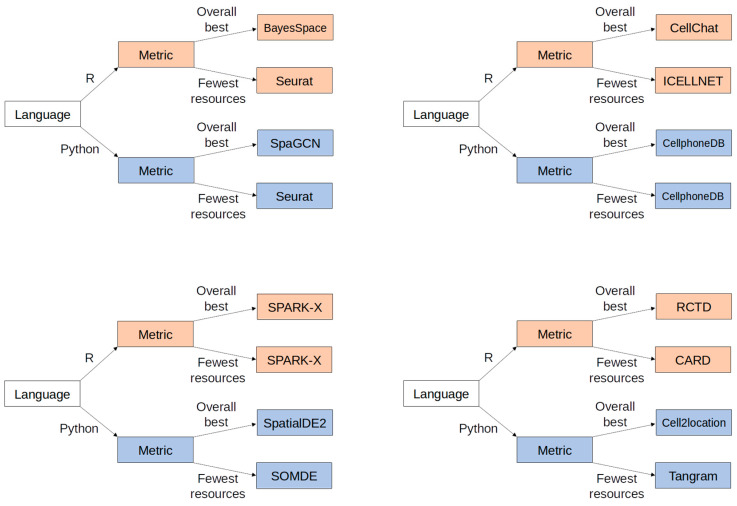
A simple decision tree to assist with determining software choices for ST analysis.

To support the decision-making for ST analysis software, we present a summary of multiple benchmarking results in a few concise formats below. First, the Figure 1 graphs provide a visual comparison of the accuracy and resource score for the top-performing software in each benchmarking paper. To calculate the resource score, runtime and RAM requirements were first normalized per study to fit a 0–1 scale. Then, the normalized runtime and RAM values were averaged together per software package per study. Finally, this average was subtracted from 1 so that larger values correspond to shorter runtimes and less RAM usage (i.e., a “better” score). Hence, the software closer to the top right corner might be more desirable because it provides higher accuracy while using fewer resources. Second, Table 5 shows more details from each benchmarking paper reviewed here so that our software comparison results can be understood in the context, e.g., the computer environment used to evaluate software in each benchmarking paper. Finally, based on these benchmarking results, Figure 2 shows a final decision tree that provides an at-a-glance recommendation for an initial or exploratory analysis.

## 7. Conclusions

The emerging field of spatial transcriptomics combines gene expression with spatial information to provide a detailed look into the workings of a given tissue sample. Within the ST analysis process, a wide variety of software is available, with each having unique traits that may affect the accuracy, time, and required computing resources. Multiple groups have performed benchmarking studies to tease out the differences between the available software packages to help researchers make informed choices for their analysis pipeline. Here, these benchmarking results were gathered and analyzed to further narrow the software choices to ones most likely to fit well into an ST analysis workflow.

Some key hurdles for the field of ST analysis to overcome were noted during the generation of this review. First, an issue mentioned in nearly all benchmarking literature was that of software performance being highly correlated with and dependent upon the dataset under analysis. The potential sample possibilities are highly diverse, so providing accurate and consistent analyses on a wide breadth of tissues will be needed. As the authors noted, this phenomenon points to the possibility of overfitting algorithms, which can lead to unreliable results. Second, and perhaps related, as postulated by some of the authors, is the lack of annotated reference data available. Some tissues have no matching reference available (at least in a matching species), or the reference has no cell type annotations, or datasets for tissue are broken into smaller anatomical regions that do not cover the entire tissue under study. While reference databases such as Aquila [90], STOmics DB [91,92], and the Spatial transcriptOmics Analysis Resource (SOAR) [93] have emerged, they do not yet offer fully annotated data that is easily usable in an ST analysis pipeline. A catalog of well-annotated cell types with expression data, perhaps selectable by organ/tissue type, would be immensely helpful in furthering ST research.

While this review aimed to be thorough, there were limitations to the research presented. First, the ST field, still being new, is constantly evolving. New methods, software options, analysis types, and workflows are published on a monthly basis, which limited the scope of information provided in this review to a snapshot of resources available at the time of writing. As noted, even within the papers reviewed, the results varied based on the software, noted particularly in the results lists returned by SVG discovery and CCC analysis. More analyses and additional data can lead to the refinement of these software packages as relationships between data output and biological reality are better understood.

Second, the list of available ST software is quite large and could not be covered comprehensively. This review was limited to current benchmarking publications as a means to allow for direct software comparison and was therefore limited to options tested in such a manner. Other options may have been overlooked in the current review, as they have not yet found their way into a benchmarking study. There is also the potential for bias introduced by those who conducted the studies having a preference for certain software packages. This has been somewhat mitigated by using multiple benchmarking studies where available and recommending software that consistently performed well across multiple studies.

Finally, this was a literature-based summary and proposal that lacked benchmarking of its own. For optimal recommendations, each proposed software package should be integrated into a pipeline and studied both as an individual step and in the process as a whole. Changing parameters or algorithms used early in the pipeline may affect the results of later steps. Ideally, the proposed pipelines would be benchmarked against several datasets and each other on a variety of platforms to draw definitive conclusions, which remains as future work.

## Figures and Tables

**Table 1:** Summary of software in tissue architecture identification benchmark studies.

Author	Software		Accuracy Metric	Dataset	Technology	Computer Environment
Cheng et al.	BayesSpace (v1.00) DR.SC (v2.9) Giotto-H (v1.0.3) Giotto-HM (v1.0.3) Giotto-KM (v1.0.3) Giotto-LD (v1.0.3) Seurat-LV (v4.0.5) Seurat-LVM V (v4.0.5)	Seurat-SLM V (v4.0.5) SpaCell (v1.0.1) SpaCell-G (v1.0.1) SpaCell-I (v1.0.1) SpaGCN (v1.2.0) SpaGCN+ (v1.2.0) StLearn (v0.3.2)	ARI with annotated datasets as ground truth. Also mean and AD of ARI across replicates.	*Mouse* olfactory bulb	Spatial Transcriptomics	Information not provided
				*Mouse* kidney coronal	10x Genomics Visium V1	
				*Mouse* brain sagittal	10x Genomics Visium V1	
				*Mouse* hypothalamic preoptic	MERFISH	
				*Mouse* somatosensory cortex	osmFISH	
				*Mouse* olfactory bulb	Stereo-seq	
				*Mouse* brain cerebellum	Slide-seq	
Hu et al.	ADEPT [9] BANKSY [10] BASS [11] BayesSpace [12] CCST [13] ConGI [14] conST [15] DeepST [16] DR.SC [17] GPSA [18] GraphST [19]	PASTE [20] PASTE2 [21] PRECAST [22] SEDR [23] SpaceFlow [24] SPACEL [25] SpaGCN [4] SpatialPCA [26] SPIRAL [27] STAGATE [28] STalign [29] STAligner [30]	ARI, NMI, AMI, and HOM, with annotated and simulated datasets as ground truth.	DLPFC	10x Genomics Visium V1	Intel Xeon W-2195 CPU 2.3 GHz 36 CPU cores 256 GB DDR4 RAM Four Quadro RTX A6000 GPUs 48 GB RAM 4608 CUDA cores
				HBCA1	10x Genomics Visium V1	
				MB2SA	10x Genomics Visium V1	
				HER2BT	Spatial Transcriptomics	
				MHPC	Slide-seq V2	
				Embryo	Stereo-seq	
				MVC	STARmap	
				MPFC	STARmap	
Yuan et al.	BASS [11] BayesSpace [12] CCST [13] conST [15] GraphST [19] Leiden [31,32]	Louvain [31] SCAN-IT [33] SEDR [23] SpaceFlow [24] SpaGCN [4] STAGATE [28] StLearn [34]	NMI against annotated datasets for ground truth.	DLPFC	10x Visium	Intel Xeon E5-2683v3 2.00 GHz 14 cores 128 GB RAM NVIDIA TITAN Xp GPU 12 GB RAM
				*Mouse* embryo	Stereo-seq	
				*Mouse* primary cortex	Barista-seq	
				*Mouse* hypothalamic preoptic	MERFISH	
				*Mouse* somatosensory cortex	osmFISH	
				*Mouse* medial prefrontal cortex	STARmap	
				*Mouse* visual cortex	STARmap*	
				*Mouse* somatosensory cortex with downsampling or noise addition	Simulated_1	
					Simulated_2	
					Simulated_3	
					Simulated_4	

**Table 2:** Summary of spatially variable gene identification benchmarking papers.

Author	Software		Accuracy Metric	Dataset	Production Method/Technology	Computer Environment
Li et al.	BOOST-GP [40] GPcounts [41] Moran’s I (Squidpy v1.2.3) nnSVG (v1.2.0) scGCO (v1.1.0) Sepal (Squidpy v1.2.3) SOMDE (v0.1.7)	SpaGCN (v1.2.5) SpaGFT (v0.1.1.4) Spanve {v0.1.0) SPARK (v1.1.1) SPARK-X (v1.1.1) SpatialDE (v1.1.3) SpatialDE2 [42]	Area under the precision–recall curve for calls against simulated data ground truth.	Simulated SVGs	Produced with normal and Gaussian distributions	AMD EPYC 7H12 CPU 64 cores 1 TB RAM A100 GPU 40 GB RAM
				Simulated non-SVGs	Identity matrix	
				Breast tumor with annotation	GP mixture model, log fold change	
				DLPFC	Manual annotation	
Chen et al.	Giotto k-means [43] Giotto rank [43] MERINGUE [44] Moran’s I [45] nnSVG [46] SOMDE [47] SPARK-X [48] SpatialDE [42]		Spearman’s correlation between SVG lists returned by software.	*Mouse* embryo E12	DbiT-seq, D1	Standard virtual machine 16 OCPUs 256 GB RAM
				*Mouse* embryo E11	DbiT-seq, D2	
				*Human* osteosarcoma	MERFISH	
				*Mouse* brain cortex	seqFISH+	
				*Mouse* cerebellum	Slide-seqV1	
				*Human* kidney cortex	Slide-seqV2	
				*Mouse* hippocampus	Slide-seqV2	
				*Mouse* brain cortex	SM_Omics, D1	
				*Mouse* brain cortex	SM_Omics, D2	
				*Human* squamous carcinoma	ST	
				*Mouse* hippocampus	ST	
				*Mouse* primary motor cortex	Visium	
				*Mouse* kidney sham	Visium, D1	
				*Mouse* kidney ischemia	Visium, D2	
				*Zebrafish* melanoma	Visium	
				*Mouse* kidney sepsis	Visium	
				*Mouse* prefrontal cortex	Visium	
				*Mouse* lymph node	Visium, D1	
				*Mouse* MCA205 tumor	Visium, D2	
				*Human* prostate	Visium	
				*Human* breast cancer	Visium, D1	
				*Human* breast cancer	Visium, D2	

**Table 3:** Summary of cell–cell communication analysis benchmarking studies.

Author	Software	Accuracy Metric	Dataset	Production Method/Technology	Computer Environment
Liu et al.	CellCall (v.0.0.0.9000) CellChat (v1.0.0) CellPhoneDB (v2) CellPhoneDB (v3) Connectome (v1.0.1) CytoTalk (v4.0.11) Domino (v0.1.1) Giotto (v1.0.4) ICELLNET (v0.99.3) iTALK (v0.1.0) NATMI [65] NicheNet (v1.0.0) scMLnet (v0.1.0) SingleCellSignalR (v1.4.0) stLearn (v0.4.7)	Distance enrichment score (DES): A calculation to quantify the consistency between the expected and observed distance of ligand–receptor pairs.	*Human* pancreatic ductal adenocarcinoma	ST	AMD EPYC 7552 48 cores 566 GB RAM
			*Human* squamous cell carcinoma	Visium V1	
			*Mouse* cortex	Visium	
			*Human* heart	Visium	
			*Human* intestine	Visium	

**Table 4:** Summary of deconvolution benchmarking studies.

Author	Software	Accuracy Metric	Dataset	Production Method/Technology	Computer Environment
Li et al.	Berglund E et al. (v0.2.0) CARD (v1.0.0) Cell2location (v0.1) DestVI (s cvi-tools 0.16.0) DSTG [66] NMFReg [67] NovoSpaRc (v0.4.4) RCTD (spacexr 2.0.0) SD^2^ [68] SpaOTsc [69] SpatialDecon [70] SpatialDWLS [71] Stdeconvolve (v1.0.0) stereoscope (v.03) SpiceMix [72] SPOTlight (v0.99.0) STRIDE [73] Tangram (v1.0.3)	JSD score, RMSE, and PCC against annotated ground truth.	*Mouse* brain medial pre-optic area	MERFISH	Intel Xeon E5-2680 v3 2.50 GHz 24 cores 528 GB RAM Two Nvidia Quadro M6000 GPUs 24 GB
			*Mouse* cortex	seqFISH+	
			PDAC	ST	
			*Mouse* brain	Visium	
			*Mouse* hippocampus	Slide-seqV2	
			Olfactory bulb	Stereo-seq	
			*Zebrafish* embryo	Stereo-seq	
Yan and Sun	Cell2location [74] DestVI [75] DSTG [66] Giotto/Hypergeometric [43] Giotto/PAGEGiotto/rank [43] MIA [76] RCTD [77] Seurat [39] SpatialDecon [70] SpatialDWLS [71] Stdeconvolve [78] stereoscope [79] SPOTlight [80] STRIDE [73] Tangram [81]	RMSE, PCC, and JSD with synthetic datasets as ground truth.	*Mouse* embryo	Sci-Space	2.7 GHz 112 cores
Li et al.	Cell2location [74] DestVI [75] DSTG [66] gimVI [82] LIGER [83] NovoSpaRc [84] RCTD [77] Seurat [39] SPaOTsc [69] SpatialDWLS [71] stereoscope [79] SPOTlight [80] StPlus [85] STRIDE [73] Tangram [81]	Pearson correlation coefficient between expression vector in ground truth dataset and expression vector in the result predicted by each integration method.	*Mouse* primary visual cortex (VISp)	BARISTAseq	CPU 2.2 GHz 144 CPU cores NVIDIA Tesla K80 GPU 12 GB RAM
			*Mouse* primary visual cortex (VISp)	ExSeq	
			*Drosophila* embryo	FISH	
			*Mouse* olfactory bulb	HDST	
			*Human* MTG	ISS	
			*Mouse* primary visual cortex (VISp)	ISS	
			*Human* osteosarcoma	MERFISH	
			*Mouse* hypothalamic preoptic region	MERFISH	
			*Mouse* primary motor cortex	MERFISH	
			*Mouse* primary visual cortex (VISp)	MERFISH	
			*Mouse* somatosensory cortex	osmFISH	
			*Mouse* liver	Seq-scope	
			*Mouse* embryonic	seqFISH	
			*Mouse* gastrulation	seqFISH	
			*Mouse* hippocampus	seqFISH	
			*Mouse* cortex	seqFISH+	
			*Mouse* olfactory bulb	seqFISH+	
			*Mouse* primary motor cortex	Slide-seq	
			*Mouse* cerebellum	Slide-seqV2	
			*Mouse* hippocampus	Slide-seqV2	
			*Human* squamous carcinoma	ST	
			*Mouse* hippocampus	ST	
			*Mouse* prefrontal cortex	STARmap	
			*Mouse* visual cortex	STARmap	
			*Human* prostate	Visium	
			*Mouse* brain	Visium	
			*Mouse* breast cancer	Visium	
			*Mouse* embryo	Visium	
			*Mouse* hindlimb muscle	Visium	
			*Mouse* hippocampus	Visium	
			*Mouse* kidney	Visium	
			*Mouse* lymph node	Visium	
			*Mouse* MCA205 tumor	Visium	
			*Mouse* prefrontal cortex	Visium	
			*Mouse* primary motor cortex	Visium	
			*Zebrafish* melanoma	Visium	

**Table 5:** Summary of computing resource requirements and accuracy metrics for different software. Orange rows denote R coding language options. Blue rows denote Python coding language options. The best scoring option(s) for each metric by study are bolded.

Tissue Architecture Identification						
Cheng et al.						
Software		Dataset	Computer Environment	Time (Min)	RAM (GB)	ARI
BayesSpace (v1.00)	R	Visium with 2696–3353 cells and 31,053 genes	Information not provided	31.623	5.495	0.820
SpaGCN (v1.2.0)	Python			**<1**	**1.000**	**0.990**
Seurat (v4.0.5)	R			**<1**	1.778	0.900
**Hu et al.**						
**Software**		**Dataset**	**Computer Environment**	**Time (Min)**	**RAM (GB)**	**Average ARI**
BASS [11]	R	Visium DLFPC, HBCA1, and MB25A datasets	Intel Xeon W-2195 CPU 2.3 GHz 36 CPU cores 256 GB DDR4 RAM Four Quadro RTX A6000 GPUs 48 GB RAM 4608 CUDA cores	316.228	Data not provided	0.450
BayesSpace [12]	R			630.957	Data not provided	0.400
SpaGCN [4]	Python			**10.000**	Ran out of RAM	0.420
STAGATE [28]	Python			19.953	Data not provided	**0.500**
**Yuan et al.**						
**Software**		**Dataset**	**Computer Environment**	**Time (Min)**	**RAM (GB)**	**NMI**
BASS [11]	R	Visium DLFPC	Intel Xeon E5-2683v3 2.00 GHz 14 cores 128 GB RAM NVIDIA TITAN Xp GPU 12 GB RAM	20.000	2.5	**0.800**
BayesSpace [12]	R			41.667	8.5	0.750
SpaGCN [4]	Python			**16.667**	1.5	0.550
STAGATE [28]	Python			**16.667**	**<1**	0.500
**Spatially Variable Gene Discovery**						
**Li et al.**						
**Software**		**Dataset**	**Computer Environment**	**Time (Min)**	**RAM (GB)**	**auPRC**
SpatialDE2 [42]	Python	Simulated 100 genes and 40,000 spots	AMD EPYC 7H12 CPU 64 cores 1 TB RAM A100 GPU 40 GB RAM	**45**	16	**12.625**
SPARK-X (v1.1.1)	R			**45**	**6**	11.875
SOMDE (v0.1.7)	Python			**45**	**6**	3.500
Moran’s I (Squidpy v1.2.3)	Python			**45**	**6**	11.000
**Chen et al.**						
**Software**		**Dataset**	**Computer Environment**	**Time (Min)**	**RAM (GB)**	**Ratio Returned List to Ground Truth List SVGs**
SPARK-X [48]	R	Combination of Visium datasets with ~12,000 genes and ~200 spots	Standard virtual machine 16 OCPUs 256 GB RAM	**10**	**<1**	**0.990**
SOMDE [47]	Python			**10**	**<1**	0.950
Moran’s I [45]	Python			30	3	0.650
**Cell–Cell Communication Analysis**						
**Liu et al.**						
**Software**		**Dataset**	**Computer Environment**	**Time (Min)**	**RAM (GB)**	**Median DES**
CellChat (v1.0.0)	R	Aggregate of 15 simulated datasets	AMD EPYC 7552 48 cores 566 GB RAM	**<1**	4	**0.082**
CellPhoneDB (v2)	Python			<1	4.5	-0.037
ICELLNET (v0.99.3)	R			**<1**	**3.2**	0.039
NicheNet (v1.0.0)	R			9.167	4	-0.322
SingleCellSignalR (v1.4.0)	R			16.667	11	-0.279
**Deconvolution**						
**Li et al.**						
**Software**		**Dataset**	**Computer Environment**	**Time (Min)**	**RAM (GB)**	**PCC**
Cell2location (v0.1)	Python	Time: MERFISH *mouse* brain ~4750 cells, 135 genes PCC: Average across 5 real-world datasets	Intel Xeon E5-2680 v3 2.50 GHz 24 cores 528 GB RAM Two Nvidia Quadro M6000 GPUs 24 GB	91.050	Data not provided	0.197
Tangram (v1.0.3)	Python			**3.867**	Data not provided	0.407
CARD (v1.0.0)	R			8.950	Data not provided	**0.425**
RCTD (spacexr 2.0.0)	R			102.117	Data not provided	0.386
**Yan and Sun**						
**Software**		**Dataset**	**Computer Environment**	**Time (Min)**	**RAM (GB)**	**PCC**
Cell2location [74]	Python	Average of 3 real-world datasets	2.7 GHz 112 cores	95.000	4.000	**0.900**
Tangram [81]	Python			**<1**	**1.000**	0.800
RCTD [77]	R			4.333	2.667	0.850
**Li et al.**						
**Software**		**Dataset**	**Computer Environment**	**Time (Min)**	**RAM (GB)**	**PCC**
Cell2location [74]	Python	Time and RAM: Simulated dataset with 20,000 spots and 10,000 cells Accuracy: Average across 32 simulated datasets	CPU 2.2 GHz 144 CPU cores NVIDIA Tesla K80 GPU 12 GB RAM	Out of RAM	Out of RAM	**0.897**
Tangram [81]	Python			**28.800**	**2.500**	0.588
RCTD [77]	R			30.700	71.000	0.606

## Data Availability

Not applicable.

## Abbreviations

The following abbreviations are used in this manuscript:

NGS	Next-generation sequencing
scRNA-seq	Single-cell RNA-seq
ST	Spatial transcriptomics
SVG	Spatially variable gene
CCC	Cell–cell communication
H&E	Hematoxylin and Eosin
HVG	Highly variable gene
FDR	False discovery rate
DES	Distance enrichment score
